# Single nucleotide polymorphism analysis in interstitial cystitis/painful bladder syndrome

**DOI:** 10.1371/journal.pone.0215201

**Published:** 2019-04-11

**Authors:** Valter D. Cassão, Sabrina T. Reis, Ruan Pimenta, Marcos Lucon, Katia R. M. Leite, Miguel Srougi, Homero Bruschini

**Affiliations:** 1 Clinics Hospital, Department of Urology, University of de Sao Paulo Medical School, Sao Paulo, Brazil; 2 Laboratory of Medical Investigation (LIM 55), Department of Urology, University of de Sao Paulo Medical School, Sao Paulo, Brazil; University Medical Center Utrecht, NETHERLANDS

## Abstract

**Introduction:**

Interstitial Cystitis (IC) is a chronic condition diagnosed based on the presence of symptoms, such as suprapubic/ pelvic pain, pressure or discomfort in association with urgency and increased urinary frequency. Confusable diseases must be excluded. However, there is no objective test or marker to establish the presence of the disease. Diagnosis and patient management is often difficult, given the poor understanding of IC pathogenesis and its unknown etiology and genetics. As an attempt to find biomarkers related to IC, we assessed the association between 20 selected single nucleotide polymorphism (SNPs) with IC and pain severity.

**Objectives:**

To assess the presence of SNPs in IC patients’ blood samples and correlate them with the disease and chronic pain condition.

**Methods:**

A case-control study was conducted. We selected 34 female patients with IC diagnosed according to NIDDK criteria and 23 patients in the control group (previously healthy women with only stress urinary incontinence). IC patients were allocated into two groups according to reported chronic pain severity. We selected the following SNPs for analysis: rs1800871, rs1800872, rs1800896, rs1800471, rs1800629, rs361525, rs1800497, rs6311, rs6277, rs6276, rs6313, rs2835859, rs11127292, rs2243248, rs6887695, rs3212227, rs1799971, rs12579350, rs3813034, and rs6746030. Genotyping was performed by real-time PCR (q-PCR).

**Results:**

The polymorphic allele of SNP rs11127292 exhibited a higher frequency in subjects with IC than in controls (p:0.01). The polymorphic allele of SNP rs6311 was more frequent in patients with severe pain (p:0.03). The frequency of the wild-type allele of SNP rs1799971 was higher in patients with mild to moderate pain (p:0.04).

**Conclusion:**

The results indicated differences in SNP frequency among subjects, suggesting that SNPs could serve either as a marker of IC or as a marker of pain severity in IC patients. The study showed promising results regarding IC and polymorphism associations. These associations have not been previously reported.

## Introduction

Interstitial Cystitis (IC) or Painful Bladder Syndrome (PBS) is a debilitating chronic pelvic pain condition primarily based on symptoms of bladder-related pain, urgency, frequency and nocturia. The etiology is still unknown. Any other confounding urologic disease must be excluded [1,2]. Initially, considered a bladder-limited syndrome, focused on highly specific cystoscopic findings, this condition has recently been regarded as a systemic chronic pain syndrome with the main symptoms related to the bladder [1–6].

However, there is no established objective marker or test to define this disorder, persisting a diagnosis of exclusion. Associations with other chronic pain conditions have been commonly described, and pain has been reported as the most debilitating symptom degrading quality of life [7–10].

Given that many associated clinical conditions have been described regarding IC/PBS, it is reasonable to assume that there are probably many underlying common physiopathologic pathways among these diseases [9,10].

IC/PBS have been historically considered a heterogeneous syndrome with intricate diagnosis and a wide variety of presentations and phenotypes. Several studies have failed to identify both the etiology and disease biomarkers, in part because few genetic studies have been conducted [11–16].

Warren et al showed a 17 times higher prevalence of IC/PBS among first-degree related adult women than in the general population. These authors have previously shown a higher prevalence of the disease among monozygotic twins[17,18]. Experimental data supports the genetic influences on the etiology of IC/PBS and on the perception of symptoms, which are most likely to be polygenic [17,18].

Our goal is to evaluate the genetic variations and individual phenotypic differences related to IC/PBS and its clinical severity based on pain symptoms.

Understanding genetic associations and their relationship to the phenotype is essential for the comprehension of diseases and human variety. In light of new advances in genetic understanding and technology, we conducted a prospective case-control study focusing on single nucleotide polymorphisms (SNPs) and their possible association with IC/PBS diagnosis and severity based on pain symptoms. Single nucleotide polymorphisms are the most common genetic variant and, once functional, may be the underlying clinical variations. In this present study, we tried to reveal the relationship between the SNPs associated with IC/PBS and the individual differences in pain experienced among the IC/PBS subjects.

The identification of SNPs associated with a disease may help to explain the disease etiology and serve as a genetic biomarker.

## Methods

### Participants

Subjects in both groups provided written informed consent to participate the study and allowed their biological samples to be genetically analyzed. Approval for the study was given by the Institutional Board of Ethics (CAPPesq–Comissão de Ética para Análise de Projetos de Pesquisa) under the number 796/015.

A case-control study was conducted. Patients with IC/PBS defined according to NIDDK (National Institute of Diabetes and Digestive and Kidney Disease) criteria (S1 Table) and low-comorbid diseases were selected [19]. Fibromyalgia was excluded. Only female participants were enlisted. The control group comprised age-matched healthy females with only genuine stress incontinence and no other urinary symptoms or disease. A total of 34 cases and 23 controls were recruited. Peripheral blood samples were collected from these subjects for the genetic analysis.

S1 Table describes the patients’ clinical and demographic features.

To define low-comorbid disease, the following conditions were considered exclusion criteria: Fibromyalgia, Irritable Bowel Syndrome, Vulvodynia, Temporomandibular Dysfunction and Orofacial Pain, suspected endometriosis, symptoms of bladder outlet obstruction, genital prolapse and symptoms suspected to be related to pudendal nerve compression or pelvic floor musculature-related pain. American College of Rheumatology (ACR) questionnaires were used to exclude any patient not diagnosed with fibromyalgia at the time of the screening appointment.

The prevalence of the selected SNPs was compared among the IC and control groups. This was the primary objective.

Second, IC/PBS females were stratified into two groups according to pain severity. This secondary objective was aimed to evaluate pain severity among IC/PBS patients, irrespective of bladder lesion severity. Pain is a subjective and multifactorial symptom. Pain questionnaires were applied, in every office appointment, in order to graduate the intensity and to minimize its subjectivity. Considering its underlying genetic background and the absence of correlation of pain severity to bladder lesion findings, evaluation of polymorphisms seems to be appropriated. Bladder-related pain was observed as the most disabling symptom and was presented in every IC/PBS patient studied.

High intensity pain perception was considered either a pain score of 7 or more or a McGill pain classification as exhausting, vicious, killing, wretched, blinding or unbearable. Patients were stratified based on pain severity data obtained from the worst pain experience reported during office follow-up.

### Study-design

All enrolled females were patients referred to the Female Urologic Division of our Institution. During regular appointments, patients were screened. Following the initial appointment, the study was explained and an Informed Consent was obtained from patients interested in participating the study.

### Measures

All participants enrolled were submitted to a comprehensive medical evaluation. Routine blood and urine samples analysis, urinary ultrasonography and urodynamic evaluation were performed. Cultures were negative. Questionnaires were applied. Clinical and demographic data were considered regarding age, timing of symptoms onset, comorbidities, symptom severity, previous treatments and quality of life index. The Interstitial Cystitis Symptom Index and Problem Index [20], and The Pelvic Pain and Urgency/Frequency (PUF) Patient Symptom Scale were adapted to Portuguese-language speakers and applied to measure the symptom severity. The validated Brazilian version (Br-MPQ)- (CASTRO, 1999) of the McGill chronic pain questionnaire (MELZACK, 1975) and Visual Analog Scale (VAS) were applied as the method to evaluate pain severity [20–23].

All IC/PBS females underwent cystoscopic evaluation, with bladder biopsy and urinary cytology performed only when individually indicated, based upon cystoscopic findings. All patients underwent bladder distention under general anesthesia.

In general, the average age of IC/PBS patients was 50,5 years. All patients of the study were Brazilian and most white females. Only one patient showed Hunner’s lesion on cystoscopy, which was treated with transurethral cauterization. All other patients presented glomerulations after bladder distension.

Cystoscopy and hydrodistension under anesthesia were fundamental to the completion of NIDDK criteria and to exclude confusable diseases.

### Polymorphism selection

We selected 20 polymorphisms (SNPs), according to a PubMed database search using the terms: “single nucleotide polymorphism”, “polymorphism”, “SNP” and “pain”, “pain syndrome”, “inflammation”, “anxiety”, “depression”, “psychiatric syndrome/disease”, “fibromyalgia” “inflammatory bowel disease”, “migraine”, “headache”, “bladder”, “frequency”, “urgency”. Table 1 shows the primers selected for polymorphism genotyping.

**Table 1:** Selected polymorphisms (SNPs).

N°	SNP	Gene	Allele	Wild-Type	Polymorphic
1	rs1800871	IL10	A/G	A	G
2	rs1800872	IL10	T/G	T	G
3	rs1800896	IL10	T/C	T	C
4	rs1800471	TGFβ1	G/C	G	C
5	rs1800629	TNFα	A/G	A	G
6	rs361525	TNFα	A/G	A	G
7	rs1800497	ANKK1/DRD2	A/G	A	G
8	rs6311	HTR2A	C/T	C	T
9	rs6277	DRD2	A/G	A	G
10	rs6276	DRD2	C/T	C	T
11	rs6313	HTR2A	A/G	A	G
12	rs2835859	KCNJ6	C/T	C	T
13	rs11127292	MYT1L	C/T	C	T
14	rs2243248	IL4	G/T	G	T
15	rs6887695	IL12B	C/G	C	G
16	rs3212227	IL12B	G/T	G	T
17	rs1799971	OPRM1	A/G	A	G
18	rs12579350	ANO2	A/G	A	G
19	rs3813034	SLC6A4	A/C	A	C
20	rs6746030	SCN9A	A/G	A	G

### Analysis of SNPs

Genomic DNA was extracted from blood samples using QIAmp DNA Blood Mini Kit (QIAGEN). The SNPs were genotyped using a TaqMan SNP Genotyping Assay Kit and an ABI 7500 fast system (Applied Biosystems, CA, USA).

**Fig 1:**
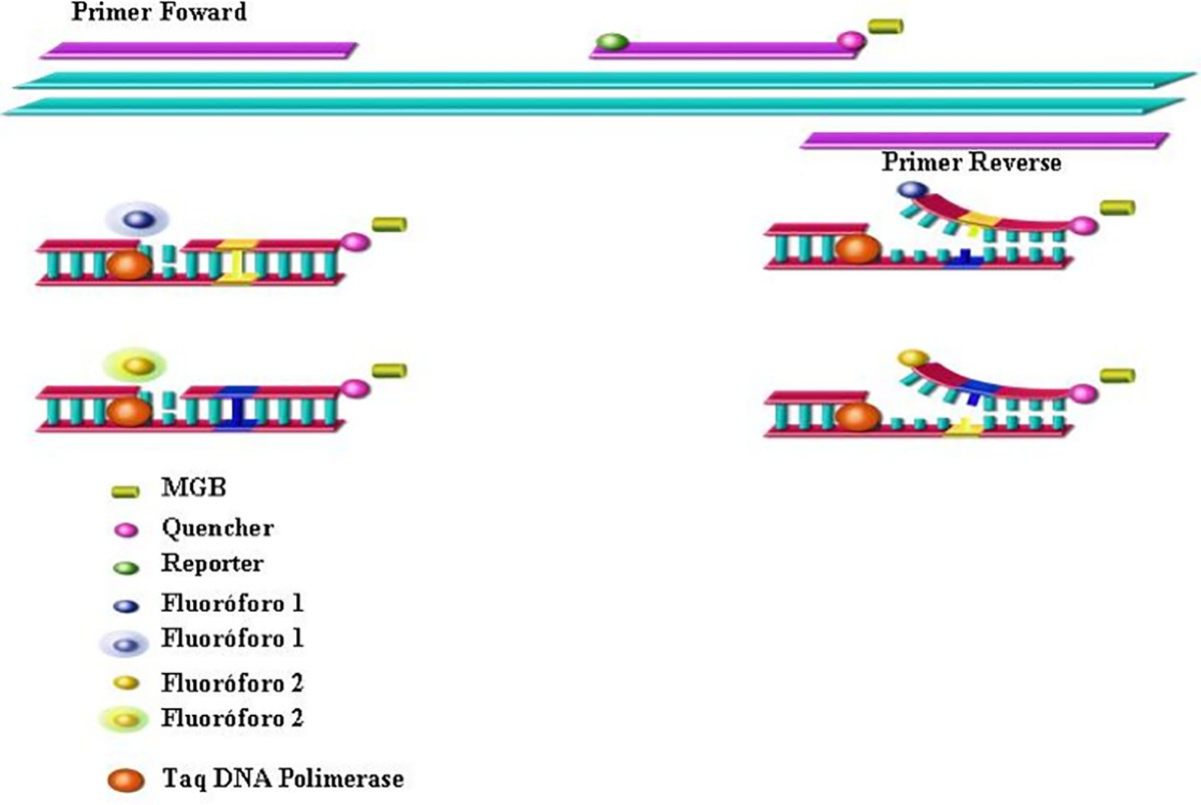
TaqMan system: TaqMan fluorogenic probes and SNP allele detection.

SNP-specific polymerase chain reaction (ss-PCR) primers (Table 2) and fluorogenic probes were designed using Primer Express (Applied Biosystems, CA, USA). The fluorogenic probes were labeled with a reporter dye (FAM or VIC) and were specific for one of the two possible bases identified for that site in the gene sequence (Fig 1).

**Table 2:** Genotype analysis comparing the IC/PBS group with the control group.

SNP	Genotype	IC/PBS group (n)	Control (n)	p
rs1800871				
	AA	5.9% (2)	4.3% (1)	0.61
	AG	85.3% (29)	78.3% (18)	
	GG	8.8% (3)	17.4% (4)	
rs1800872				
	TT	8.8% (3)	0% (0)	0.17
	TG	91.2% (31)	95.7% (22)	
	GG	0% (0)	4.3% (1)	
rs1800896				
	TT	44.1% (15)	26.1% (6)	0.31
	TC	50% (17)	60.9% (14)	
	CC	5.9% (2)	13% (3)	
rs1800471				
	GG	0% (0)	0% (0)	0.25
	GC	8.8% (3)	13% (3)	
	CC	91.2% (31)	87% (20)	
rs1800629				
	AA	0% (0)	0% (0)	0.06
	AG	76.5% (26)	95.7% (22)	
	GG	23.5% (8)	4.3% (1)	
rs361525				
	AA	0% (0)	0% (0)	0.48
	AG	55.9% (19)	65.2% (15)	
	GG	44.1% (15)	34.8% (8)	
rs1800497				
	AA	3% (1)	13% (3)	0.10
	AG	91.2% (31)	82.6% (19)	
	GG	5.8% (2)	4.3% (1)	
rs6311				
	CC	20.6% (7)	30.4% (7)	0.14
	CT	64.7% (22)	39.1% (9)	
	TT	14.7% (5)	30.4% (7)	
rs6277				
	AA	14.7% (5)	13% (3)	0.79
	AG	58.8% (20)	52.2% (12)	
	GG	26.5% (9)	34.8% (8)	
rs6276				
	CC	8.8% (3)	13% (3)	0.81
	CT	44.1% (15)	39.1% (9)	
	TT	47.1% (16)	47.9% (11)	
rs6313				
	AA	14.7% (5)	21.7% (5)	0.78
	AG	44.1% (15)	39.1% (9)	
	GG	41.2% (14)	39.1% (9)	
rs2835859				
	CC	8.8% (3)	4.3% (1)	0.79
	CT	47.1% (16)	52.2% (12)	
	TT	44.1% (15)	43.5% (10)	
rs11127292				
	CC	76.5% (26)	100% (23)	0.01
	CT	23.5% (8)	0% (0)	
	TT	0% (0)	0% (0)	
rs2243248				
	GG	0% (0)	0% (0)	0.51
	GT	91.2% (31)	95.7% (22)	
	TT	8.8% (3)	4.3% (1)	
rs6887695				
	CC	23.5% (8)	17.4% (4)	0.44
	CG	35.3% (12)	52.2% (12)	
	GG	41.2% (14)	30.4% (7)	
rs3212227				
	GG	23.5% (8)	47.8% (11)	0.14
	GT	32.4% (11)	17.4% (4)	
	TT	44.1% (15)	34.8% (8)	
rs1799971				
	AA	47.1% (16)	26.1% (6)	0.28
	AG	50% (17)	69.6% (16)	
	GG	2.9% (1)	4.3% (1)	
rs12579350				
	AA	2.9% (1)	0% (0)	0.70
	AG	17.6% (6)	17.4% (4)	
	GG	79.4% (27)	82.6% (19)	
rs3813034				
	AA	14.7% (5)	21.7% (5)	0.74
	AC	82.4% (28)	73.9% (17)	
	CC	2.9% (1)	4.3% (1)	
rs6746030				
	AA	5.9% (2)	13% (3)	0.52
	AG	20.6% (7)	26.1% (6)	
	GG	73.5% (25)	60.9% (14)	

The target sequence was amplified in a 10 μl reaction volume that contained 5 μl of Taq-Man1 Universal PCR Master Mix, 0.25 μl of SNP Genotyping Assay (primers and probes), 1 μl of genomic DNA, and 3.75 μl of DNase-free water. The PCR cycling conditions were 2 minutes at 50°C and 10 minutes at 95°C, followed by 40 cycles of 15 seconds at 95°C and 60 seconds at 60°C. After PCR amplification, an endpoint plate reading was performed using an Applied Biosystems 7500 fast Real-Time PCR System. Sequence Detection System (SDS) software uses the fluorescence measurements made during the plate reading to plot fluorescence (Rn) values based on the signals from each well. The plotted fluorescence signals indicate which alleles are in each sample.

TaqMan SNP Genotyping Assay overview, representing the two primers and the two MGB probes annealing specifically to a complementary oligonucleotide sequence presented in the polymorphism. Each probe is specific to a given SNP allele (polymorphic and wild-type allele). Each probe end has an attached molecule, a fluorescent molecule (dye reporter) at one end, and a non-fluorescent molecule at the other (dye quencher). When the probe is intact, the proximity of the quencher dye to the reporter dye suppresses the reporter fluorescence. During the PCR cycles, Taq DNA polymerase exonuclease activity cleaves only the probes hybridized to the target, separating the reporter dye from the quencher dye, increasing the fluorescence of the reporter. (Source: Reis ST, Applied Biosystems user guide).

### Statistical analysis

Statistical methods were performed using SPSS 19.0. Univariate analysis was done using chi-square test (χ^2^) comparing the difference between the patients and controls. Statistical significance was considered at p < 0.05 (95% CI—confidence interval).

Polymorphisms were studied in all 34 IC/PBS patients and 23 controls. Prevalence of the 20 selected SNPs was assessed among the two groups.

Once measurable, the strength of the association comparing the polymorphisms was estimated by calculating the odds ratio (OR).

## Results

PCR products from the 20 selected SNPs were detected in all DNA specimens from 34 IC/PBS patients and 23 controls. Genotypes and allele distribution were compared.

The genotype results are presented in Table 2. Considering SNP rs11127292, of the 34 IC/PBS patients, 76.5% (26) had a wild-type homozygote genotype (CC), and 23.5% (8) were the heterozygote genotype (CT). All control subjects were the wild-type homozygote genotype. No subject in the study were the polymorphic homozygote genotype. Thus, we found a statistically significant difference compared with the controls (Pearson chi-square p = 0.01).

The allele distribution is presented in Table 3. All controls (100% (N = 23)) carried the wild-type allele (C) of SNP rs11127292, and none carried the polymorphic allele (T). In contrast, in IC/PBS, 76.5% (N = 26) of females had the wild-type allele (C), and 23.5% (N = 8) had the polymorphic allele (T). (p = 0.01). The difference in polymorphic allele (T) presence was statistically significant between IC/PBS and controls (Pearson chi-square p = 0.01).

**Table 3:** Statistical association comparing allele prevalence among the IC/PBS group and the control group.

SNP	Allele	IC/PBS (n)	Control (n)	Odds Ratio (OR)	p
rs1800871					
	A*	5.9% (2)	4.3% (1)	0.72 [0.06–8.52]	0.79
	G	94.1% (32)	95.7% (22)		
rs1800872					
	T*	8.8% (3)	0% (0)	–	0.14
	G	91.2% (31)	100.0% (23)		
rs1800896					
	T*	44.1% (15)	26.1% (6)	0.44 [0.14–1.41]	0.16
	G	55.9% (19)	73.9% (17)		
rs1800471					
	G*	0% (0)	0% (0)	–	–
	C	100.0% (34)	100.0% (23)		
rs1800629					
	A*	0% (0)	0% (0)	–	–
	G	100.0% (34)	100.0% (23)		
rs361525					
	A*	0% (0)	0% (0)	–	–
	G	100.0% (34)	100.0% (23)		
rs1800497					
	A*	2.9% (1)	13.0% (3)	4.50 [0.48–50.89]	0.14
	G	97.1% (33)	87.0% (20)		
rs6311					
	C*	20.6% (7)	30.4% (7)	1.68 [0.50–5.69]	0.39
	T	79.4% (27)	69.6% (16)		
rs6277					
	A*	14.7% (5)	13.0% (3)	0.87 [0.18–4.06]	0.85
	G	85.3% (29)	87.0% (20)		
rs6276					
	C*	8.8% (3)	13% (3)	1.35 [0.27–6.84]	0.70
	T	91.2% (31)	87% (20)		
rs6313					
	A*	14.7% (5)	21.7% (5)	1.61 [0.40–6.35]	0.49
	G	85.3% (29)	78.3% (18)		
rs2835859					
	C*	8.8% (3)	4.3% (1)	0.47 [0.04–4.81]	0.51
	T	91.2% (31)	95.7% (22)		
rs11127292					
	C*	76.5% (26)	100.0% (23)	–	0.01
	T	23.5% (8)	0.0% (0)		
rs2243248					
	G*	0% (0)	0% (0)	–	–
	T	100.0% (34)	100.0% (23)		
rs6887695					
	C*	23.5% (8)	17.4% (4)	0.68 [0.18–2.60]	0.57
	T	76.5% (26)	82.6% (19)		
rs3212227					
	G*	23.5% (8)	47.8% (11)	2.97 [0.95–9.30]	0.06
	T	76.5% (26)	52.2% (12)		
rs1799971					
	A*	47.1% (16)	26.1% (6)	0.39 [0.12–1.25]	0.11
	G	52.9% (18)	73.9% (17)		
rs12579350					
	A*	2.9% (1)	0% (0)	–	0.40
	G	97.1% (33)	100.0% (23)		
rs3813034					
	A*	14.7% (5)	21.7% (5)	1.61 [0.40–6.35]	0.49
	C	85.3% (29)	78.3% (18)		
rs6746030					
	A*	5.9% (2)	13.0% (3)	2.40 [0.36–15.64]	0.34
	G	94.1% (32)	87.0% (20)		

* wild-type allele

IC/PBS females were further allocated into two subgroups according to pain severity symptoms (McGill and VAS). Group 1 comprised 12 females with mild to moderate pain and group 2 comprised 21 IC patients with severe pain. The genotypes and allele distribution were compared and presented in Table 4 and Table 5, respectively. A total of 33 IC/PBS patients were recruited. One patient missed the follow-up and did not participate this second part of the study.

**Table 4:** Genotype analysis comparing the mild to moderate pain group and the severe pain group among IC/PBS patients.

SNP	Genotype	Mild/Moderate Pain (n)	Severe Pain (n)	p
rs1800871				
	AA	8.3% (1)	4.8% (1)	0.13
	AG	74.7% (9)	95.2% (20)	
	GG	16.6% (2)	0% (0)	
rs1800872				
	TT	16.7% (2)	4.8% (1)	0.25
	TG	83.3% (10)	95.2% (20)	
	GG	0% (0)	0% (0)	
rs1800896				
	TT	33.3% (4)	47.6% (10)	0.14
	TC	50% (6)	52.4 (11)	
	CC	16.7% (2)	0% (0)	
rs1800471				
	GG	0% (0)	0% (0)	0.70
	GC	8.3% (1)	9.5% (2)	
	CC	91.7% (11)	90.5% (19)	
rs1800629				
	AA	0% (0)	0% (0)	0.94
	AG	75% (9)	76.2% (16)	
	GG	25% (3)	23.8% (5)	
rs361525				
	AA	0% (0)	0% (0)	0.26
	AG	41.7% (5)	61.9% (13)	
	GG	58.3% (7)	38.1% (8)	
rs1800497				
	AA	0% (0)	4.8% (1)	0.39
	AG	100% (12)	85.7% (18)	
	GG	0% (0)	9.5% (2)	
rs6311				
	CC	41.7% (5)	9.5% (2)	0.08
	CT	50% (6)	71.4% (15)	
	TT	8.3% (1)	19% (4)	
rs6277				
	AA	16.7% (2)	14.3% (3)	0.17
	AG	41.7% (5)	71.4% (15)	
	GG	41.7% (5)	14.3% (3)	
rs6276				
	CC	16.6% (2)	4.8% (1)	0.08
	CT	58.4% (7)	38.1% (8)	
	TT	25.0% (3)	57.1% (12)	
rs6313				
	AA	8.3% (1)	19% (4)	0.35
	AG	33.3% (4)	47.6% (10)	
	GG	58.3% (7)	33.3% (7)	
rs2835859				
	CC	16.7% (2)	0% (0)	0.15
	CT	41.7% (5)	52.4% (11)	
	TT	41.7% (5)	47.6% (10)	
rs11127292				
	CC	66.7% (8)	81% (17)	0.36
	CT	33.3% (4)	19% (4)	
	TT	0% (0)	0% (0)	
rs2243248				
	GG	0% (0)	0% (0)	0.90
	GT	91.7% (11)	90.5% (19)	
	TT	8.3% (1)	9.5% (2)	
rs6887695				
	CC	16.7% (2)	28.6% (6)	0.37
	CG	25% (3)	38.1% (8)	
	GG	58.3% (7)	33.3% (7)	
rs3212227				
	GG	8.3% (1)	33.3% (7)	0.24
	GT	41.7% (5)	42.9% (9)	
	TT	50% (6)	23.8% (5)	
rs1799971				
	AA	25% (3)	61.9% (13)	0.06
	AG	75% (9)	33.3% (7)	
	GG	0% (0)	4.8% (1)	
rs12579350				
	AA	0% (0)	4.8% (1)	0.72
	AG	16.7%(2)	19% (4)	
	GG	83.3% (10)	76.2% (16)	
rs3813034				
	AA	16.7% (2)	14.3% (3)	0.74
	AC	83.3% (10)	81% (17)	
	CC	0% (0)	4.8% (1)	
rs6746030				
	AA	8.3% (1)	4.8% (1)	0.52
	AG	8.3% (1)	23.8% (5)	
	GG	83.3% (10)	71.4% (15)	

**Table 5:** Allele prevalence analysis comparing the mild to moderate pain group to the severe pain group among IC/PBS patients.

SNP	Allele	Mild/Moderate Pain (n)	Severe Pain (n)	Odds Ratio (OR)	p
rs18008971					
	A*	8.3% (1)	4.8% (1)	1.81 [0.10–31.99]	0.67
	G	91.7% (11)	95.2% (20)		
rs1800872					
	T*	16.7% (2)	4.8% (1)	4.00 [0.32–49.59]	0.25
	G	83.3% (10)	95.2% (20)		
rs1800896					
	T*	33.3% (4)	47.6% (10)	0.55 [0.12–2.40]	0.42
	G	66.7% (8)	52.4% (11)		
rs1800471					
	G*	0% (0)	0% (0)	-	-
	C	100.0% (12)	100.0% (21)	-	-
rs1800629					
	A*	0% (0)	0% (0)	-	-
	G	100.0% (12)	100.0% (21)	-	-
rs361525					
	A*	0% (0)	0% (0)	-	-
	G	100.0% (12)	100.0% (21)		
rs1800497					
	A*	0% (0)	4.8% (1)	1.05 [0.95–1.15]	0.44
	G	100.0% (12)	95.2% (20)		
rs6311					
	C*	41.7% (5)	9.5% (2)	6.78 [1.06–4.36]	0.03
	T	58.3% (7)	90.5% (19)		
rs6277					
	A*	16.7% (2)	14.3% (3)	1.00	0.85
	G	83.3% (10)	85.7% (18)	1.20 [0,17 – 8,42]	
rs6276					
	C*	16.7% (2)	4.8% (1)	4.44 [0.35–55.57]	0.21
	T	83.3% (10)	95.2% (20)		
rs6313					
	A*	8.3% (1)	19.0% (4)	0.38 [0.03–3.92]	0.40
	G	91.7% (11)	81.0% (17)		
rs2835859					
	C*	16.7% (2)	0% (0)	0.83 [0.64–1.07]	0.06
	T	83.3% (10)	100.0% (21)		
rs11127292					
	C*	66.7% (8)	81.0% (17)	0.47 [0.09–2.38]	0.35
	T	33.3% (4)	19.0% (4)		
rs2243248					
	G*	0% (0)	0% (0)	-	-
	T	100% (12)	100% (21)		
rs6887695					
	C*	16.7% (2)	28.6% (6)	0.50 [0.08–2.99]	0.44
	T	83.3% (10)	71.4% (15)		
rs3212227					
	G*	8.3% (1)	33.3% (7)	0.18 [0.01–1.70]	0.10
	T	91.7% (11)	66.7% (14)		
rs1799971					
	A*	25.0% (3)	61.9% (13)	0.20 [0.04–0.99]	0.04
	G	75.0% (9)	38.1% (8)		
rs12579350					
	A*	0% (0)	4.8% (1)	1.05 [0.95–1.15]	0.44
	G	100.0% (12)	95.2% (20)		
rs3813034					
	A*	16.7% (2)	14.3% (3)	1.20 [0.17–8.42]	0.85
	C	83.3% (10)	85.7% (18)		
rs6746030					
	A*	8.3% (1)	4.8% (1)	1.81 [0.10–31.99]	0.67
	G	91.7% (11)	95.2% (20)		

* wild-type allele

Genotype distribution failed to show any significant difference between the two groups.

Data considering SNP rs6311 revealed a significant increase in the prevalence of the polymorphic T allele in group 2 patients (severe pain). The polymorphic allele was presented on 90.5% of these cases, while it was only in 58.3% of group 1 patients (mild to moderate pain)–(p = 0.03)–OR: 6.78 (IC: 1.06–43.36).

Regarding SNP rs1799971, there was a significant increase in the prevalence of the polymorphic G allele in group 1. The polymorphic allele occurred in 75% of mild to moderate pain patients, while it was only in 38.1% of severe pain patients (group 2)—(p = 0.04)—OR:0.2 (IC:0.04–0.99).

## Discussion

This study attempted to correlate SNPs to IC/PBS and to pain severity in these patients. Associations were found in three of the 20 tested SNPs. One of them, rs11127292, was associated with the disease itself. Of the other two, rs6311 was associated with the cases with severe pain, and rs1799971 with mild to moderate pain patients.

In regard to the IC/PBS patients, the statistically significant finding was an increase in the prevalence of SNP rs11127292 polymorphic T allele. Although none of the subjects had the homozygote polymorphic genotype, 23.5% of the cases presented one copy of the T allele, while no control subject presented the polymorphic allele. Interestingly this allele was associated with the disease but not pain symptom severity in this study.

This SNP was previously studied in patients with fibromyalgia. Polymorphism rs11127292 is located at the MYT1L (myelin transcription factor 1-like) gene and was associated with worse symptomatology in fibromyalgia patients [24]. This gene encodes a protein linked to the neuronal pathways and that has been associated with neuronal differentiation. Polymorphisms in this gene are described in the literature in association with neuropsychiatric disorders and fibromyalgia. These findings suggest a role of the central nervous system in genetic susceptibility to IC/PBS [24]. In this study patients were selected based on a low-comorbidity basis, to reduce bias due to overlapping diseases. In this way fibromyalgia was an exclusion criterion.

Based on this finding, we can suggest that both IC/PBS and FM may have common underlying nociceptive mechanisms. SNP rs11127292 may also be related to pain in FM and IC/PBS subjects independently, since this study revealed a positive association to IC/PBS in non-fibromyalgic females, and literature previously associated this SNP to FM.

The literature supports the idea that some genetic variations in serotoninergic pathways may be associated with chronic pain syndrome susceptibility [25]. SNPs in the serotonin gene receptor (5HT2AR gene) are related to FM and vestibulodynea [26,27]. SNP rs6311 is a polymorphism of the 5HT2AR gene. In this study, rs6311 was not associated with IC/PBS compared to the controls, but it was associated as a marker of pain severity in IC/PBS. The polymorphic T allele was present in 90.5% of patients with severe pain, demonstrating a significantly increased prevalence compared that in patients with mild to moderate pain. The polymorphic allele of SNP rs6311 was a significant marker of susceptibility to severe pain in IC/PBS, increasing the risk of presenting worse pain by more than six times.

There is no previous study in the literature associating rs6311 with IC/PBS. However, some data suggest that the prevalence of the polymorphic allele of this SNP is increased in patients with dyspareunia and concomitant body pain, which gives additional support to our findings [26,27].

These findings suggest that the previously described serotoninergic involvement in chronic pain syndromes may influence pain perception and susceptibility in IC/PBS patients. Future studies with other polymorphisms of serotonergic pathways can confirm this association. In contrast to this finding, the frequency of another polymorphism of the serotonin receptor gene (5HT2AR), rs6313, was also assessed in our study and showed neither a significant difference in prevalence between patients with IC and controls nor a significant difference among patients with IC who presented with more or less pain symptoms.

Previous studies correlate SNP rs1799971 with pain, due to its association with opioid consumption and its high prevalence in a wide variety of ethnicities, reaching 10–15% in Caucasians and up to 50% in Asians. SNP rs1799971 is a polymorphism of OPRM1, a gene which encodes a receptor linked to pain susceptibility, as well as sensitivity to opioids, both endogenous and exogenous [28–30].

In the present study, mild to moderate pain IC/PBS patients had a wild-type A allele frequency of 25% and polymorphic G allele frequency of 75%. IC/PBS females with severe pain had a wild-type A allele prevalence of 61.9% and polymorphic G allele prevalence of 38.1%. These data point to a wild-type A allele association to increased pain sensitivity, suggesting a possible protective effect of the polymorphic G allele against pain susceptibility in these patients.

Supporting our findings, previous studies showed an association of SNP rs1799971 G polymorphic allele to an increased affinity of opioid receptors to endogen β endorphins. However, this allele is also associated with reduced μ opioid receptor (OPRM1 gene encoded) expression and signaling. Data in the literature suggest that the G polymorphic allele plays a protective role against pain and is associated with a need for higher doses of opioid drugs for pain relief due to the reduced expression and signaling of the μ opioid receptor [28–30].

It is important to emphasize that although some studies suggest an association between SNP rs1799971 G allele and pain protection, in a meta-analysis, this association with pain perception was not significant, leading only to the need for slightly lower opioid drug doses for pain relief in those carrying this polymorphic allele [30].

Genetic trials assessed polymorphisms and mutations of the SCN-9A gene. This gene encodes a voltage-gated sodium channel (Nav1.7) expressed in the peripheral sensory neurons and dorsal root ganglia that have a critical role in pain sensitivity. It is considered that mutations and polymorphisms in the SCN-9A gene may be related to variations in pain perception [31–33]. A single study demonstrated an increased prevalence of the wild-type homozygous SNP rs6746030 (SCN9A) genotype and heterozygous genotype in IC/PBS patients (39.6%) compared with that in the controls (11.5%) [31].

In this present study, this finding was not corroborated since there was no statistical difference in this SNP both when comparing IC/PBS patients to the controls and when comparing pain sensitivity among the IC/PBS patients.

Recently, some studies have used a less restricted definition of IC/PBS, based on the American Urological Association (AUA) guidelines and European Society for the Study of Interstitial Cystitis (ESSIC), allowing a larger and more heterogeneous number of patients to engage in these studies. The association of IC/PBS with chronic pain syndromes is evident and there is an increased assumption of IC/PBS as a chronic systemic pain syndrome focused on the bladder [8, 9, 34, 35].

In this study, we applied the most restrict, specific and objective criteria for the definition of IC/PBS, allowing a group of selected homogeneous females with a lower range of somatic pain, other than that focused on the bladder.

In terms of starting an earlier treatment, there was a recent attempt in clinical practice to extend the definition of some chronic pain syndromes, such as FM, IBS, and IC/PBS, which facilitate the diagnosis and increases the prevalence of the associations of these conditions due to overlapping symptoms.

Pain is probably the most debilitating symptom of IC/PBS patients worldwide. In this study, all 34 selected patients experienced this symptom, and in most of the cases it was classified as severe pain (64% of IC/PBS females). It can be inferred that IC can represent a particular chronic pain syndrome phenotype with underlying genomic susceptibilities and variations in common. An additional limiting factor to the study is that although our analysis yielded statistically significant results if these were subjected to multiple-test statistical correction by Bonferroni method, p-value adjusted for the number of SNPs tested in the study would not be significant.

Although this study had a relatively small sample size, we demonstrated the genetic variation in IC/PBS females, however, the differences in SNP expressions might result from the heterogeneity of this syndrome. A clear understanding of genetics along with the polymorphisms that may be related to IC/PBS pathophysiology and that may influence pain susceptibility can help elucidate this syndrome and provide a promising contribution to its diagnosis and management.

## Conclusion

SNP rs11127292 was more prevalent in the IC/PBS group than in the control group (p = 0.01).

The SNP rs6311 polymorphic allele was more prevalent in the severe pain IC/PBS group, and the SNP rs1799971 polymorphic allele was more prevalent in patients with mild to moderate pain.

Results must be cautiously considered. Although patients were meticulously selected based on rigid research criteria (NIDDK), IC/PBS is still considered a rare condition, with a difficult diagnosis and heterogeneous phenotypes, which result in a limited sample size, particularly when genetics and polymorphism analysis are concerned. This relatively small sample size may be responsible for the lack of statistically significant associations. Genetic composition can vary with the studied population. This study was conducted in a nationwide referral center of IC/BPS but limited to females and a single country with a heterogeneous genetic profile in the population. Further analyses, including more patients and studies with different ethnic populations, are required to corroborate the relevance of these associations.

## Ethics statement

Subjects in both groups provided written informed consent to participate the study and allowed their biological samples to be genetically analyzed. Approval for the study was given by the Institutional Board of Ethics (CAPPesq–Comissão de Ética para Análise de Projetos de Pesquisa) under the number 796/015.

## Supporting information

S1 Table Interstitial cystitis and control group patients characteristics. (DOCX)
