# Supplementary material for: Single nucleotide polymorphism analysis in interstitial cystitis/painful bladder syndrome
Source: PLoS One. 2019 Apr 11;14(4):e0215201. doi: 10.1371/journal.pone.0215201 (PMC6459590; doi:10.1371/journal.pone.0215201)
Supplement: S1 Table — (DOCX) [file pone.0215201.s001.docx]

**Supporting information** – Supplementary table.

**S1 Table. Interstitial Cystitis and Control Group Patients Characteristics**

| IC Group | AGE  (years) | RACE | O’ Leary- Sant | PUF | PAIN  (VAS) | Hunner | Comorbidities | BMI |
| --- | --- | --- | --- | --- | --- | --- | --- | --- |
| 1 | 48 | White | 28 | 25 | 8 | - | - | 27.4 |
| 2 | 35 | White | 20 | 20 | 6 | - | + | 29.3 |
| 3 | 42 | White | 31 | 30 | 9 | - | - | 31.1 |
| 4 | 59 | White | 30 | 27 | 9 | - | + | 27.4 |
| 5 | 37 | White | 28 | 25 | 9 | - | - | 29.3 |
| 6 | 41 | White | 19 | 18 | 4 | - | - | 28.6 |
| 7 | 50 | White | 21 | 16 | 6 | - | - | 31.2 |
| 8 | 68 | White | 17 | 15 | 4 | - | - | 29.6 |
| 9 | 48 | Non-white | 20 | 20 | 5 | - | - | 27.8 |
| 10 | 52 | White | 24 | 21 | 5 | - | - | 26.8 |
| 11 | 47 | White | 18 | 16 | 5 | - | + | 29.2 |
| 12 | 69 | White | 18 | 15 | 6 | - | - | 30.4 |
| 13 | 56 | Non-white | 29 | 32 | 9 | - | - | 26.1 |
| 14 | 62 | White | 18 | 15 | 4 | - | - | 27.7 |
| 15 | 52 | White | 24 | 23 | 7 | - | + | 28.3 |
| 16 | 47 | White | 19 | 17 | 5 | - | - | 28.6 |
| 17 | 65 | White | 24 | 26 | 7 | - | - | 30.3 |
| 18 | 31 | White | 30 | 25 | 7 | - | - | 28.0 |
| 19 | 43 | White | 33 | 30 | 9 | - | + | 27.5 |
| 20 | 67 | White | 26 | 23 | 7 | - | - | 27.3 |
| 21 | 70 | White | 18 | 15 | NA | - | - | 29.4 |
| 22 | 60 | Non-white | 19 | 20 | 4 | - | + | 25.3 |
| 23 | 53 | White | 18 | 21 | 4 | - | - | 27.9 |
| 24 | 61 | White | 27 | 23 | 9 | - | - | 27.2 |
| 25 | 49 | White | 29 | 25 | 9 | - | - | 28.7 |
| 26 | 46 | White | 19 | 16 | 7 | + | - | 29.0 |
| 27 | 45 | White | 23 | 19 | 7 | - | + | 27.5 |
| 28 | 32 | White | 13 | 22 | 7 | - | - | 28.8 |
| 29 | 62 | White | 29 | 25 | 7 | - | - | 27.6 |
| 30 | 64 | White | 33 | 33 | 9 | - | + | 29.8 |
| 31 | 51 | Non-white | 30 | 27 | 8 | - | - | 26.7 |
| 32 | 37 | White | 32 | 30 | 9 | - | - | 27.1 |
| 33 | 31 | White | 27 | 26 | 8 | - | + | 26.2 |
| 34 | 67 | White | 22 | 18 | 7 | - | - | 26.4 |

| Control  Group | AGE  (years) | RACE | O’ Leary- Sant | PUF | PAIN  (VAS) | Hunner | Comorbidities | BMI |
| --- | --- | --- | --- | --- | --- | --- | --- | --- |
| 1 | 51 | White | - | - | 0 | - | - | 27.3 |
| 2 | 64 | Non-white | - | - | 0 | - | - | 28.6 |
| 3 | 53 | White | - | - | 0 | - | - | 30.1 |
| 4 | 60 | White | - | - | 0 | - | - | 26.6 |
| 5 | 47 | White | - | - | 0 | - | - | 27.8 |
| 6 | 68 | Non-white | - | - | 0 | - | - | 28.9 |
| 7 | 55 | White | - | - | 0 | - | - | 31.0 |
| 8 | 53 | White | - | - | 0 | - | - | 26.5 |
| 9 | 65 | White | - | - | 0 | - | - | 25.6 |
| 10 | 54 | Non-white | - | - | 0 | - | - | 27.4 |
| 11 | 61 | White | - | - | 0 | - | - | 32.2 |
| 12 | 63 | White | - | - | 0 | - | - | 28.7 |
| 13 | 68 | White | - | - | 0 | - | - | 29.7 |
| 14 | 57 | White | - | - | 0 | - | - | 29.5 |
| 15 | 52 | White | - | - | 0 | - | - | 26.4 |
| 16 | 51 | White | - | - | 0 | - | - | 27.8 |
| 17 | 73 | White | - | - | 0 | - | - | 32.3 |
| 18 | 49 | White | - | - | 0 | - | - | 26.2 |
| 19 | 66 | White | - | - | 0 | - | - | 27.0 |
| 20 | 57 | White | - | - | 0 | - | - | 26.8 |
| 21 | 68 | White | - | - | 0 | - | - | 27.9 |
| 22 | 53 | White | - | - | 0 | - | - | 27.5 |
| 23 | 56 | White | - | - | 0 | - | - | 28.1 |
